# A scoping review of Chinese national policies to promote the three-medicals linkage reform since 2014

**DOI:** 10.1038/s44401-025-00012-9

**Published:** 2025-03-28

**Authors:** Tingzhuo Liu, Jiarong Shen, Xinyi Zhang, Shangzhi Xiong, Pengpeng Ye, Wenlan Dong, Yongchen Wang, Wei Jiang, Maoyi Tian

**Affiliations:** 1 School of Public Health, Harbin Medical University, Harbin, China; 2 School of Laboratory Medicine and Biotechnology, Southern Medical University, Guangzhou, China; 3 The George Institute for Global Health, Faculty of Medicine and Health, University of New South Wales, Sydney, NSW Australia; 4 National Centre for Non-Communicable Disease Control and Prevention, Chinese Centre for Disease Control and Prevention, Beijing, China; 5 Department of General Practice, The Second Affiliated Hospital of Harbin Medical University, Harbin, China; 6 Chinese Centre for Disease Control and Prevention, Beijing, China

**Keywords:** Health policy, Health services, Public health

## Abstract

In 2014, for the first time, the Chinese government included the “three-medicals linkage” as a key task of medical reform, aiming to integrate medical care, medical insurance, and medicine into a cohesive health governance and service delivery model. This study reviews national policies on the “three-medicals linkage” issued from 2014 to 2023 to analyze its framework, evolution, strengths, and challenges. A thematic synthesis of 121 policy documents, selected from an initial 4392 records from State Council and its affiliated ministries, revealed 12 major strategies, including “reform of medical insurance payment methods” and “universal medical insurance and informatization of medical insurance”. But areas such as health information and workforce development remain underemphasized. Although the “three-medicals linkage” has strengthened the health system, gaps remain, particularly in multi-sectoral coordination and information integration. We recommend enhancing multi-sectoral cooperation, addressing deficiencies in health information and workforce, and establishing robust monitoring, evaluation, and incentive mechanisms.

## Introduction

In April 2009, China unveiled an extensive and intricate healthcare reform plan, pledging to provide equitable, high-quality, and affordable basic medical services and healthcare coverage for all citizens by 2020^1,2^. Before this, access to healthcare in China was largely determined by individuals’ ability to pay, due to inadequate government funding and low medical insurance coverage^1^. The 2009 reforms prioritized equity in healthcare services, focusing on medical care, medicine, and medical insurance as key areas of the reform^3^. By 2013, healthcare insurance coverage had reached over 95% of the Chinese population and has remained stable since^4^. Although the reforms improved access to and utilization of medical services, there remains gaps in addressing the efficiency in healthcare service delivery^5^. These inefficiencies arose from factors such as lack of multi-sectoral coordination, and inappropriate incentive measures (e.g., allowing a 15% markup on prescription drugs and linking doctors’ compensation to the profits of medical institutions)^6,7^. In response to these challenges, the Chinese government introduced the concept of the “Linkage of Medical Care, Medicine, and Medical Insurance (three-medicals linkage)” as a key task in the national healthcare reform agenda in 2014^8^. This strategy aims to achieve comprehensive reform by integrating medical care, medicine, and medical insurance.

Since 2000, China’s “three-medicals linkage” reform has undergone two stages. The first stage (2000–2014) is mainly based on the pilot programs led by the local government. During this period, China successfully established a nationwide disease prevention and control system, as well as a public health emergency response system. Essential public health services were provided free of charge to all urban and rural residents^9^, and by 2013, the national health insurance coverage rate increased to over 95%^1^. At the first stage of the reform, the pilot programs were only implemented in selected municipals, including Shanghai (since 2002) and Sanming from Fujian province (since 2012). The key components of the pilot program consisted of adjusting medical service costs, lowering drug prices and limiting the expenditures of the health insurance^10^. The second stage began in 2014, when the central government elevated the “three-medicals linkage” reform to a national strategy, making it a key task of the national healthcare reform^11^. In 2016, the “Healthy China 2030” policy set the “three-medicals linkage” as a long-term goal for the healthcare system^12^. Despite the efforts of the Chinese government to promote the reform of “three-medicals linkage”, there are still many challenges in the process of policy formulation and implementation^13,14^.

This study aims to conduct a comprehensively review of China’s national policies to understand the development progress of the “three-medicals linkage” policy strategy, with five specific objectives. First, to assess the number and types of relevant policies related to the “three-medicals linkage” and explore the main path of policy-making. Second, to determine the leading policies and policy evolution process. Third, to identify the key areas of the “three-medicals linkage” from the health system perspective. Fourth, to explore the key implementation strategies of the reform. Lastly, to identify the strengths and potential gaps in the policy strategies to inform future policy-making related with “three-medicals linkage”.

## Results

### Policy search process and results

**Fig. 1:**
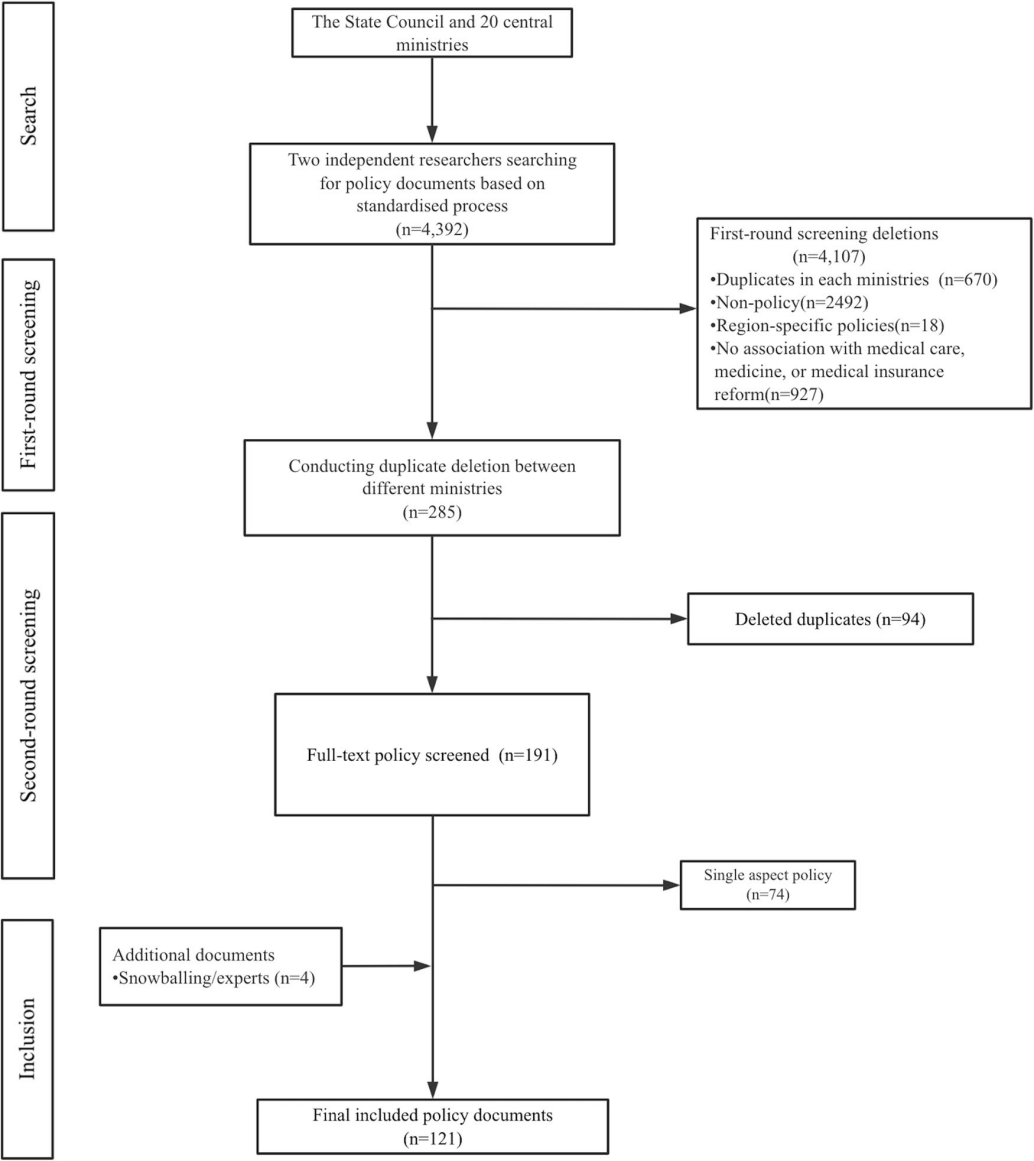
Flow chart. The initial search yielded 4,392 records. After two rounds of screening, a total of 121 policy documents were included for analysis.

In the preliminary search, we identified a total of 4392 records issued by The State Council and 20 subordinate ministries (Fig. 1). In the first round of screening, we excluded 4107 records based on the inclusion and exclusion criteria. Most of the excluded records were non-policy documents, such as news articles, reports, and patent announcements, as well as policies unrelated to medical care, medicine, and medical insurance (*n* = 927). After removing duplicated records (*n* = 670) within the same ministry and policies not at the national level (*n* = 18), 285 records remained for the second round of screening. In this round, we excluded duplicate records between different ministries (*n* = 94), resulting in 191 policy documents for full-text screening. During the full-text screening, we excluded policies that only addressed one single component of the “three-medicals linkage”, i.e., medical care, medicine, or medical insurance (*n* = 74). In addition, we identified four more records through snowball searches and consultations with policy experts. Finally, 121 policy documents were included in the analysis.

### Policy development stages

**Fig. 2:**
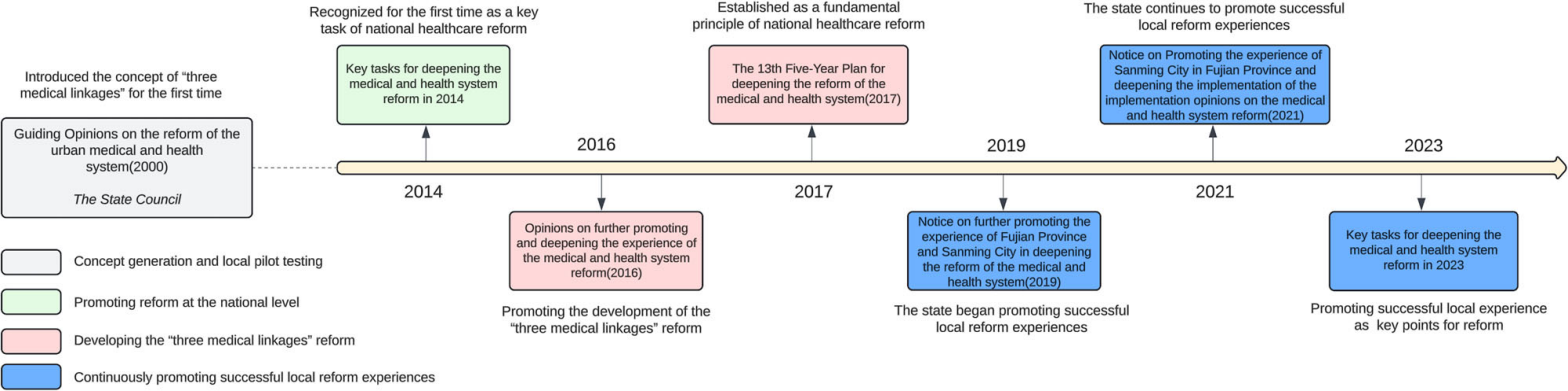
Leading policies and policy evolution. Colors represent different stages of policy development in the reform process.

The development of China’s “three-medicals linkage” reform can be categorized into four distinct stages (Fig. 2).The first stage began in 2000 with the introduction of the “three-medicals linkage” concept. During this period, the reform was largely driven by local governments through pilot programs, without formal promotion at the national level. The second stage commenced in 2014 when the State Council officially identified the “three-medicals linkage” as a key focus of national healthcare reform. This marked the beginning of nationwide efforts to implement the policy. In the third stage, starting in 2016, the State Council further emphasized the reform as a critical element of deepening the healthcare system restructuring. The “three-medicals linkage” reform was incorporated into China’s 13th Five-Year Plan as part of the country’s long-term development goals. Since 2019, the reform entered its fourth stage. The government began promoting the successful practices of the “Sanming model” (Box 1) as a template for replication across the country, aiming to scale up these local achievements nationwide.

Box 1 China’s Sanming model The Sanming healthcare reform was initiated due to the risk of collapse of the urban workers’ medical insurance fund, with losses exceeding 200 million Yuan by 2011. In 2012, under Vice Mayor Zhan Jifu, Sanming launched a comprehensive reform encompassing three crucial areas.

Firstly, the governance structure was reformed to integrate the management of three medical insurance schemes (for urban residents, rural residents, and civil servants) and establish a medical insurance management center to link medicine, insurance, and hospitals, serving as a national demonstration. Secondly, the drug procurement process was unified with a “two-invoices mechanism,” allowing only two invoices in the wholesale chain and centralizing procurement to reduce prices and eliminate intermediaries. These reforms received support from the State Council. Thirdly, Sanming altered physician compensation by delinking income from unmonitored profits (medical rebates) that made up over 80% of total income, replacing it with a higher basic salary plus performance-based bonuses. Before the Sanming reform, the doctors’ salary was composed by two parts, one-third from a basic salary, while the remaining two-thirds were based on performance bonuses tied to profits, including sales revenue from drugs and equipment. The Sanming reform decoupled doctors’ income from profits, linking it instead to factors such as professional title, the number of patients served, and the quality of care provided. This reform did not lead to significant staff turnover, with fewer than 40 doctors leaving public hospitals annually over the past 5 years.

The Sanming model successfully reduced medical costs while maintaining clinical quality. Since 2014, the comprehensive reforms led to a 6.1% reduction in the medical care cost per outpatient visit and a 15.4% reduction per inpatient admission^17^. A significant contributor to these reductions was the decrease in drug expenditure, which dropped by approximately 29% per outpatient visit and 53% per inpatient admission^17^. Additionally, the model reduced the proportion of hospital revenue generated from drug sales from 47% to 26%^17^. In addition to reducing the costs of drugs and medical devices, the Sanming reform increased income from medical services by adjusting service prices, leading to an optimized hospital revenue structures. As a result of these cost-saving measures, the medical insurance fund improved from a deficit of 200 million Yuan to a surplus of 130 million Yuan, indicating that the financial balance of Sanming’s medical insurance system shifted from negative to positive.

### Multi-sectoral collaboration in policy making

**Fig. 3:**
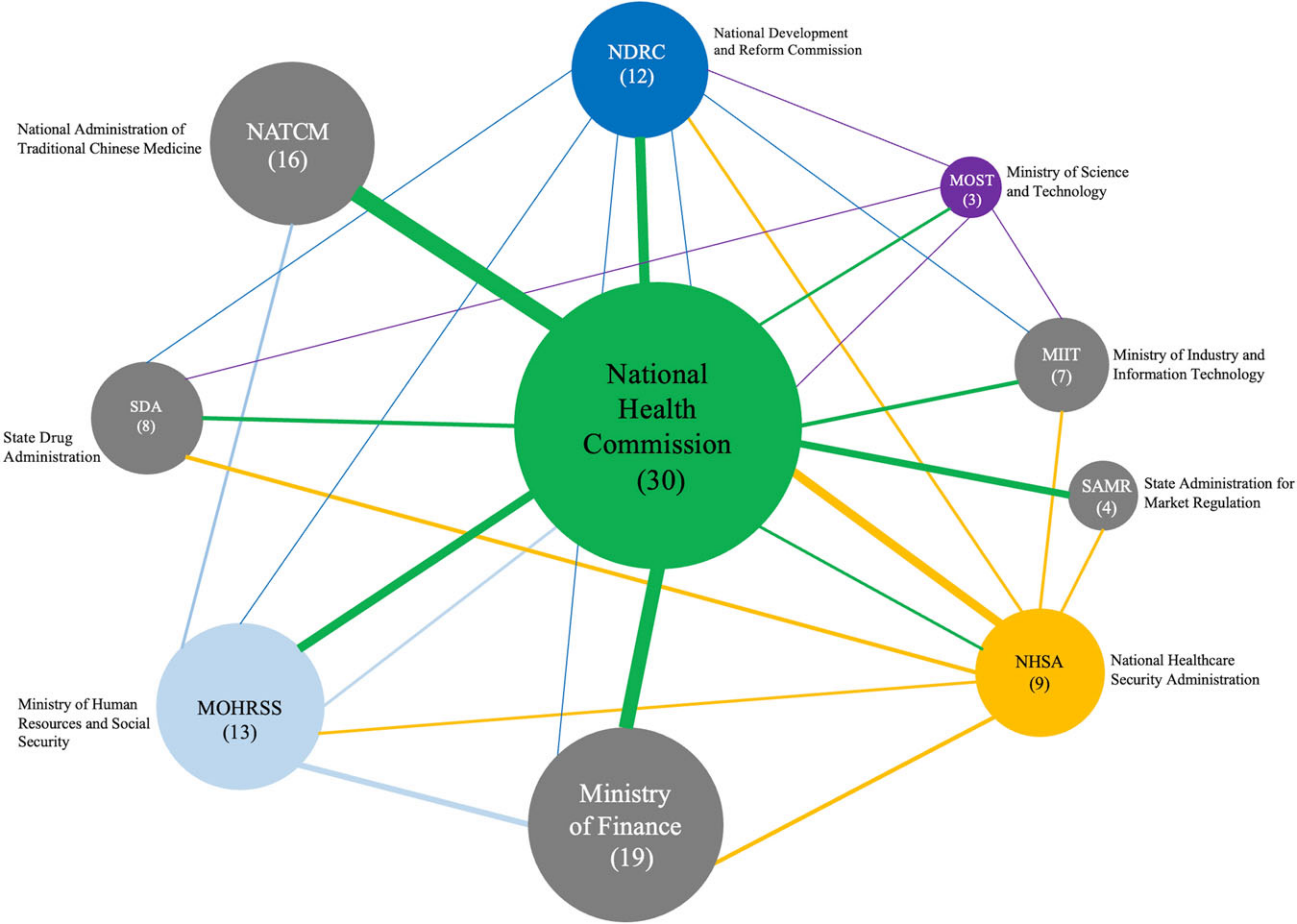
The network plot of ministries involved in the co-developed policies. The numbers in parentheses indicate the number of policy documents developed by the respective department. The thickness of the lines and size of the circle is proportional to the number of times that ministries were involved in co-develop policies. The colored circles are the ministries that have issued the co-developed policy as the lead, and the ministries in the gray circles are only involved in the co-developed policy.

Approximately 30% of the “three-medicals linkage” reform policies were jointly issued by at least two ministries (*n* = 36, 29.7%). National Health Commission of China, formerly the Ministry of Health, was the lead agency in multi-sectoral policy formulation (*n* = 32), followed by the Ministry of Finance (*n* = 23) and the Ministry of Human Resources and Social Security (*n* = 17) (Fig. 3). Other ministries include the National Administration of Traditional Chinese Medicine (*n* = 16), the National Healthcare Security Administration (*n* = 13), the National Development and Reform Commission (*n* = 12), the State Drug Administration (*n* = 10), and the Ministry of Industry and Information Technology (*n* = 7). Notably, the National Health Commission, Ministry of Human Resources and Social Security, National Development and Reform Commission, National Healthcare Security Administration, and Ministry of Science and Technology are the leading ministries in formulating policies, while other ministries mainly play a participating role.

### Major policy strategies for “three-medicals linkage” in the four linkage modes

**Fig. 4:**
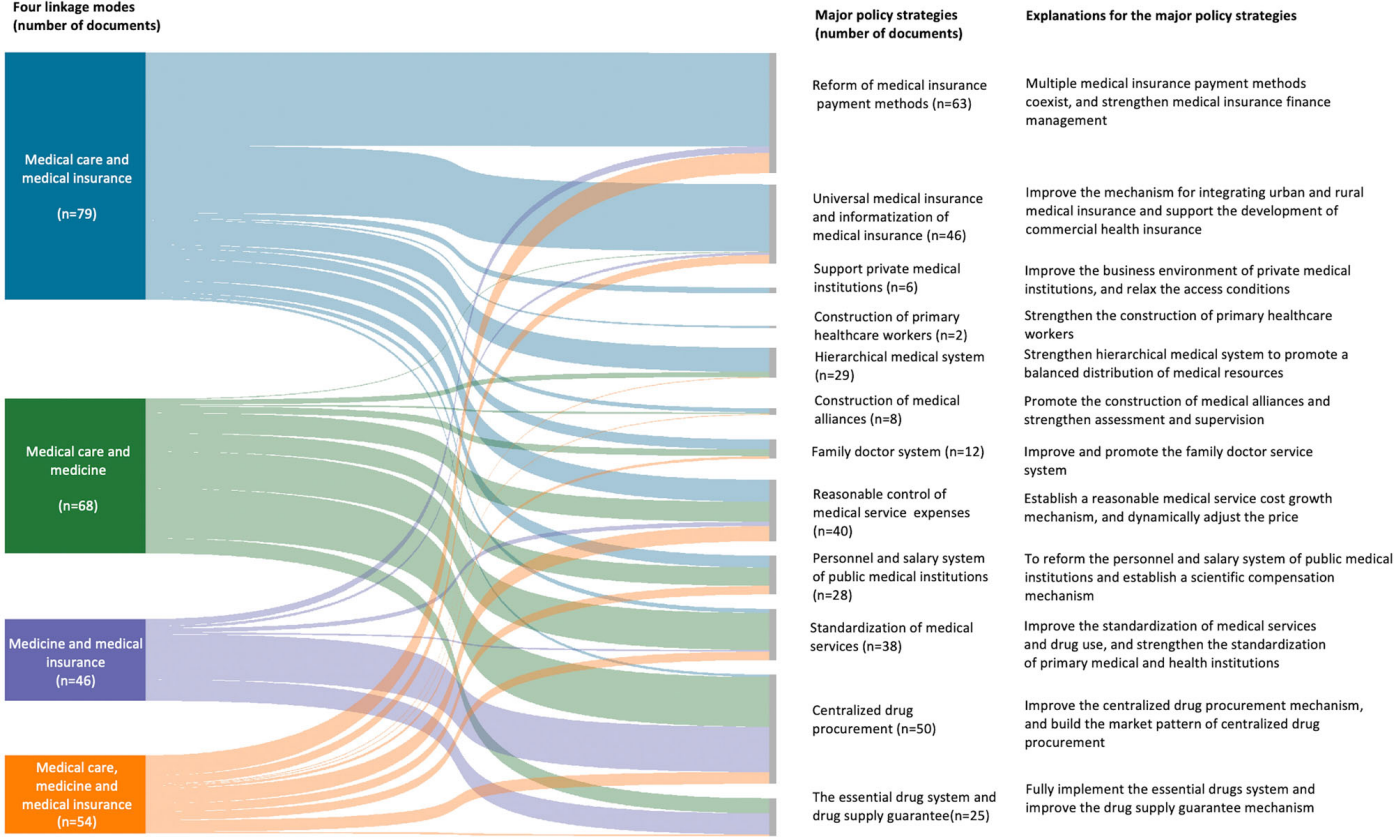
Distribution of the main strategies of “three-medicals linkage” in the four linkage modes. The numbers in parentheses indicate the number of policy documents. Colors represent different linkage modes. Each of the major policy strategies is explained in detail below.

Through inductive coding of 121 policy documents, we identified 12 major policy strategies (Fig. 4). Of these documents, 79 included measures for the “medical care—medical insurance” linkage mode, followed by “medical care—medicine” (*n* = 68), “medical care—medicine—medical insurance” (*n* = 54), and “medicine—medical insurance” (*n* = 46). The 12 policy strategies derived from multiple policy documents cover one or more linkage modes. The top three strategies were the “reform of medical insurance payment methods” (mentioned in 63 policy documents, 55%), “centralized drug procurement” (*n* = 50, 44%), and “universal medical insurance and informatization of medical insurance” (*n* = 46, 40%).

### The distribution of linkage patterns in WHO six building blocks

**Fig. 5:**
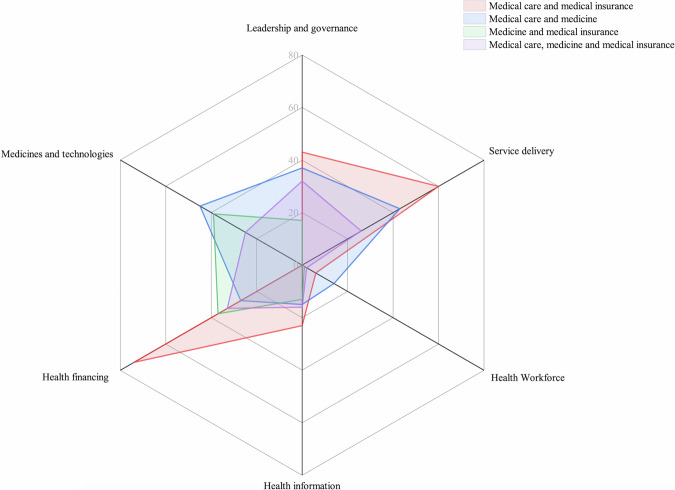
The specific measures of the “three-medicals linkage” policies from 2014 to 2023. The numbers represent the number of policy documents in each WHO’s six building blocks of health system component and the four linkage models.

The policy documents related to the four linkage modes covered nearly all of the WHO’s health system building blocks, though the extent of coverage varied across each mode. Most linkage modes primarily focused on health financing, leadership, and governance, with the “medical care—medical insurance” mode having the highest number of policy measures. However, there was limited attention given to health information systems, and particularly to the health workforce (Fig. 5). The distributions of the polices benchmarked to the WHO’s six health system building blocks from 12th Five Year Plan to 14th Five Year Plan are provided in Supplementary Fig. 2.

## Discussion

This scoping review comprehensively examined the national policies and key strategies related to the “three-medicals linkage” reform, since the Chinese central government designated it as a key task of healthcare reform in 2014. Our analysis highlighted the Chinese government’s continuous efforts in promoting this reform, and identified twelve major policy strategies. Guided by the WHO’s framework, we found uneven policy efforts across different health system’s building blocks.

In the policy-making process for China’s “three-medicals linkage” reform, two unique approaches complement the traditional top-down pathway. The first approach involves annual updates and continuous adjustments. Each year, the Chinese government issues the *Key Tasks of Deepening the Reform of the Medical and Health System*, a policy document from the State Council that outlines the reform’s evolving priorities. This allows policymakers to make dynamic adjustments, ensuring that the reform remains aligned with emerging healthcare needs and broader health system goals. By continuously refining the reform’s focus, this method promotes deeper and more comprehensive changes over time. The second approach involves leveraging successful local reforms as models for nationwide implementation. China’s government actively encourages local innovation in policy areas, creating a fertile environment for practical solutions to emerge from regional governments. Once proven effective, these local innovations are scaled up to a national policy^15^. The experience of Sanming City in Fujian Province, where significant progress was achieved under the “three-medicals linkage” reform, exemplifies this process. Sanming’s successes were recognized by the central government, leading to the wider adoption of its strategies as a blueprint for reform across the country^11^. Together, these approach illustrate how China’s “three-medicals linkage” reform balances top-down direction with grassroots innovation, fostering both adaptability and the replication of best practices. This combination ensures that policy development is both responsive and scalable, enabling the reform to meet the complex challenges of the healthcare system.

The “three-medicals linkage” reform involves multiple areas including medical care, medicine, and medical insurance, and requires close collaboration between multiple government departments, for example, the Health Commission and Health Security Administration, during the policy formulation and implementation. Broader participation from various government departments would facilitate policy coordination and bridge policy gaps^16^. For example, in the Sanming reform, the municipal government reoriented the management structure, with the aim to strengthen the connections and further eliminate the fragmentation between the departments. Specifically, the authority to formulate health related policies and manage public hospitals was centralized into a committee, chaired by the Vice Mayor of Sanming City. This allowed the government to establish a coherent set of goals for health reform and enforce health administrators accountable for their performance. This committee was also responsible for strategic planning, for example, overseeing primary healthcare infrastructure development, equipment and drug procurement, and hospital physicians’ compensation methods. This integration laid the foundation for effective implementation of complex reforms^17^.

Among the specific measures of the “three-medicals linkage” policy, the number of policy documents containing both medical care and medical insurance measures is the largest. These measures are primarily distributed among the main strategies of “reforming medical insurance payment methods”, “universal medical insurance and informatization of medical insurance”, and the “hierarchical medical system”. However, only a few policy measures have achieved full coordination across medical care, medicine, and medical insurance. Medical insurance policies play a dominant role in the “three-medicals linkage” reform, linking medical insurance with medical care through payment method reforms and guiding the reasonable allocation of medical resources. The wide uptake of medical insurance has improved the accessibility of medical care, increased the utilization of medical services^18–20^, and boosted outpatient visits^21^. However, this focus on improving accessibility has not been matched by equivalent improvements in the quality of health care. For example, “three-medicals linkage” policy is set to encourage patients to seek care at the primary healthcare institutions, patients often bypass primary healthcare institutions primarily because of the weak service capacity and the shortage of essential drugs^22^. This also results in lower service satisfaction with the primary health care providers^23^. Future medical insurance policies shall fully consider the coordination with medical care and medicine related policy, strengthening the clinical capacity of primary healthcare institutions, ensuring the continuous availability of essential medicines, and establishing effective incentive mechanisms for primary healthcare providers.

“Centralized drug procurement” measures account for a significant proportion of the “three-medicals linkage” policy strategies. While the rationale to promote centralized drug procurement is to lower drug prices, prior studies indicated that this reform has not significantly reduce inpatient medical expenses^24,25^. This is because the lower profit of drugs included in centralized procurement leads to an increase in the use of substitute drugs (those not centrally procured)^26^. This might be explained that the pricing reform is not accompanied by changes in the incentive plans for medical service providers, particularly in public hospitals^27^. Medical service providers are, therefore, inclined not to prescribe^26^. Integrating medicine policies with medical care and medical insurance, such as establishing tiered pricing for essential drugs in combination with the medical insurance payment system, needs to be well considered. For example, a multi-tiered drug pricing system might be established to classify drug prices based on their clinical necessity and cost-effectiveness. Additionally, different reimbursement rates can be applied to centrally procured and non-centrally procured drugs, with adjustment of current medical insurance policies, to encourage healthcare institutions and patients to prioritize the selection of cost-effective medications^28^.

The “three-medicals linkage” reform plays a pivotal role in advancing health equity and bridging the urban-rural divide in access to healthcare resources. Evidence suggests that systematic reforms strengthening primary healthcare, can significantly enhance primary healthcare utilization regardless of the residential locations, contributing to a reduction in urban-rural health disparities^29^. Addressing the health workforce gap and providing advanced professional training for primary healthcare providers have the potential to improve the efficiency of primary healthcare institutions, thereby expanding rural populations’ access to high-quality healthcare services^30^. Furthermore, family doctor systems have been shown to substantially improve residents’ quality of life, with individuals enrolled in such programs experiencing better outcomes compared to their counterparts without access^31^, and further narrow the gaps in healthcare accessibility between urban and rural areas^31^. Nevertheless, pronounced health inequities persist, largely driven by imbalanced allocation of healthcare resources^32^. To address these disparities, future iterations of the “three-medicals linkage” reform should prioritize enhancing the remuneration of primary healthcare workers, recalibrating resource distribution between urban and rural regions, and ensuring that differences in medical expenditure burdens are maintained with an equity lens.

From the perspective of health system, a notable feature of national policies related to China’s “three-medicals linkage” reform is that it attaches great importance to health financing, service delivery, leadership and governance of the WHO six building blocks. In particular, health financing plays a dominant role in this reform. China has proactively promote the Diagnosis Related Groups and Diagnosis-Intervention Packet payment schemes over the past 5 years^33,34^. Although preliminary evaluations found that these alternative payment schemes were associated with reduction in total medical expenditures, out-of-pocket costs and length of stay^35–40^, but assessment on healthcare quality were insufficient and with mixed findings^35,38–42^. Whereas, health information and health workforce, are rarely addressed in the “three-medicals linkage” reform policy. Although information development has been identified as a key priority in the national 13th Five-Year Plan, the current fragmented health information system is difficult to realize the effective information exchange between medical care, medicine and medical insurance. Similarly, despite recent studies have identified health workforce shortages in both quantity and quality as major challenges, current policies are insufficient to address these longstanding issues^23,43,44^.

To the best of our knowledge, this is the first scoping review of national policies for China’s “three-medicals linkage” reform. A significant strength of this study is the use of qualitative approach to guide the content analysis of policy documents. However, this study has several limitations. First, we could only include open-source policy documents publicly available on the Chinese central government websites. Although we attempted to reduce the omission of significant policy documents through snowball searches and expert consultations, further research is needed to discover potentially unpublished relevant policies. Second, this study focuses on central government policies and does not consider local uptake, adaptation, and innovation at the provincial level or below. Future research is needed to explore how these central policies translate into local policies at different jurisdictional levels and how local governments can innovate in the policy-making process. Finally, the data extracted in this review are insufficient to assess the implementation and effectiveness of these policies.

Since 2014, China’s steadfast political commitment to the “three-medicals linkage” reform has been encouraging. We propose four recommendations for future policy formulation and implementation. First, to encourage multi-sectoral coordination in future development of “three-medicals linkage” related policies. Specifically, to establish high-level organizational leadership and independent coordination institutions to create cross-sectoral decision-making mechanisms. Second, to develop integrated health information systems, incorporating standardized data variables, data structures, and data management systems to allow routine and effective monitoring and evaluation. Third, to empower the capacity of healthcare workers, particularly primary healthcare providers, through rigorous training, assessment, and incentive mechanisms. Finally, to develop context-tailored monitoring, evaluation and incentive mechanisms of the “three-medicals linkage”, ensuring such reform is consistent with the intended goals.

## Methods

### Study design

Guided by the PRISMA extension for Scoping Reviews (PRISMA-ScR Supplementary Table 1)^45^, this study reviewed all open-accessed national-level policy documents, related to the “three-medicals linkage” in China since 2014, when the State Council announced the “three-medicals linkage” as a key task of medical reform^8^. The study protocol is registered on the Open Science Framework platform (10.17605/OSF.IO/E4SYV).

### Connotations of medical care, medicine and medical insurance

“Medical care” includes public health service and medical service, across all health institutions at all levels (primary, secondary and tertiary levels). “Medicine” consists of the production, circulation, distribution and security system of drugs, consumables, instruments and other products used in medical and health care activities. “Medical insurance” mainly refers to the medical security system, including basic medical insurance, medical assistance and various forms of supplementary insurance.

### Theoretical frameworks

**Fig. 6:**
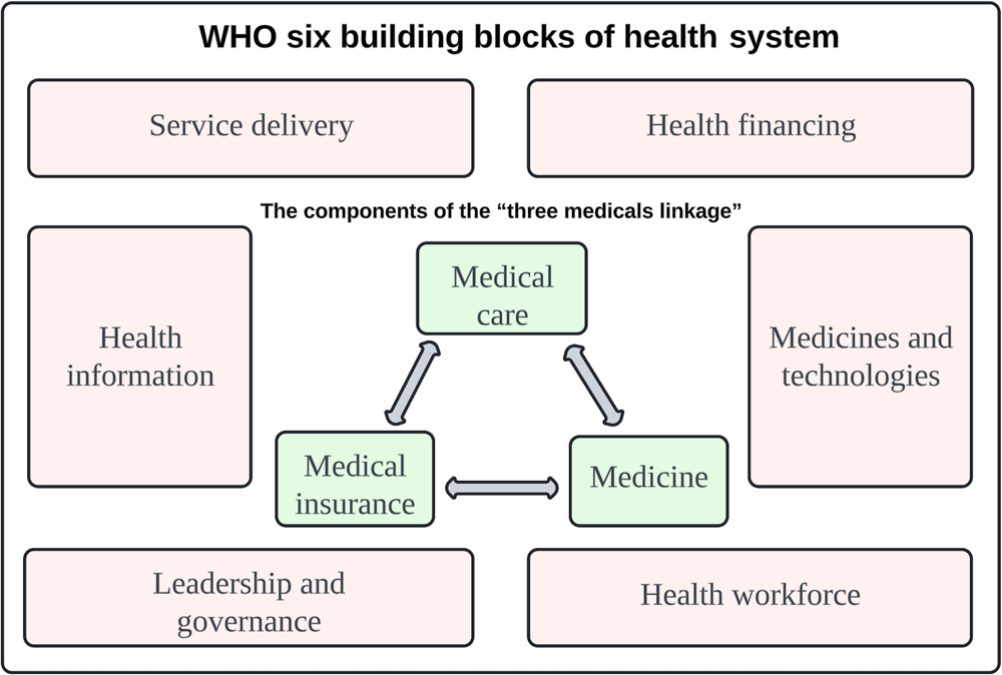
The analytical framework, comprising the WHO’s six building blocks and the “three-medicals linkage”. The theoretical framework adapted in this study consists of two components: the WHO’s six building blocks of health system and the four linkage modes of the “three-medicals linkage”.

The theoretical framework adapted in this study consists of two components (Fig. 6). First, the WHO six building blocks framework explain the concept of the six interrelated components of the health system (e.g., service delivery, health workforce) and describe the “constitute” of the health system and it was used to examine how China’s central policy documents have promoted the reform of the “three-medicals linkage” reform from these six aspects. Second, the interrelation among the three components of the “three-medicals linkage” (i.e., medical care, medicine, and medical insurance) constitutes the connotation of the “three-medicals linkage” reform, which entails four linkage modes by nature, including medical care and medicine linkage mode, the medical care and medical insurance linkage mode, the medicine and medical insurance linkage mode, and the medical care, medicine and medical insurance linkage mode.

### Data source

In January 2024, we conducted online searches through the publicly accessible official websites of the State Council of China and its subordinate ministries at the national level (Supplementary Table 2). Subordinate ministries were included based on the functions designated by the State Council, encompassing those directly related to healthcare reform (such as the National Health Commission) and those that might influence healthcare reform (such as the Ministry of Education).

### Search strategy and selection criteria

Due to the heterogeneity of the search function on the websites of the ministries, it was difficult to perform an uniformed search. The search strategy was divided into the following three steps. First, we used one of the four Chinese keywords to perform separate search on the websites of each ministry, including *San Yi Lian Dong* (“three-medicals linkage”), *San Ming Yi Gai* (Sanming Healthcare Reform), *San Ming Gai Ge* (Sanming reform), *Yiliao- Yiyao- Yibao* (medical care, medicine and medical insurance), reviewed and consolidated the search records. Second, we conducted a snowball search of the references in the policy and/or the other cited policies to supplement the search results. Finally, we further consulted policy experts from the Chinese Center for Disease Control and Prevention to avoid major omissions of the relevant policies.

At the end of the search, one researcher (TL) removed all duplicated search records. Subsequently, two researchers (TL, JS) screened and excluded non-policy documents. The remaining policy documents were then independently screened by two researchers (TL, JS), with discrepancies resolved through group discussions involving other senior researchers (MT, PY, SX, XZ). Policy documents usually do not have summaries or abstracts and were screened by applying the following inclusion and exclusion criteria. We included policy documents related to the “three-medicals linkage” reform, issued by the designated national ministries since The State Council took the “three-medicals linkage” as a key task of medical reform in 2014, and contained two or three aspects of the “three-medicals linkage”, including medical care, medicine and medical insurance. The search spanned from May 13th, 2014^8^, when The State Council’s official release of “*Key Tasks for Deepening the reform of the Medical and Health System*”, to December 31st, 2023.

### Data extraction

For each policy document included in the analysis, we extracted information of the policy title, the corresponding ministry or ministries released the policy, release date, and details about the cross-referenced policy documents (Supplementary Table 3). Policies were classified based on whether they were individually released by a single ministry or jointly issued by multiple ministries. The frequency of each ministry’s participation in joint releases was used as an indicator of the ministry’s involvement in multi-sectoral collaboration in policy development. Regarding the release time, each policy document is further divided into three periods: 2014–2015, 2016–2020 and 2021–2023, representing the 12th, 13th and 14th five-year plan of the China’s central government, but acknowledging that the 12th and 14th five-year plans are not fully covered. Cross-referencing information was extracted from the introduction section of each policy, which typically details how each policy refers to others for guidance or supporting evidence.

### Policy content analysis

Two researchers (TL and JS) conducted a thematic analysis of the included policy documents, guided by the theoretical framework of the WHO six building blocks and the linkage modes of the “three-medicals linkage” (Fig. 6). Both deductive and inductive coding methods were used.

To identify key areas of the “three-medicals linkage” from a health system perspective, we employed a deductive coding approach. Specific action measures from the policies were extracted and systematically assigned to the WHO six building blocks and the four linkage modes. There might be multiple action measures within one policy document. Each action measure was coded into the corresponding blocks or modes. For example, one policy document addressing the improvement of the standardization level of drug use in medical institutions, containing policy action measures related to “medical care” and “medicine”. These measures were then coded into the “medical care and medicine linkage mode” in the four linkage modes and the “service delivery” and “medicines and technologies” blocks in the WHO six building blocks accordingly. Detailed coding book was available from Supplementary Table 4.

To identify policy planning and implementation strategies related to the “three-medicals linkage”, we applied an inductive coding approach. First, specific strategies or action items from the policy documents were identified and coded as sub-themes. Second, these sub-themes were then synthesized into higher-level main themes grouping multiple related sub-themes. These main themes, referred as “major strategies”, represent the fundamental planning and implementation strategies of China’s “three-medicals linkage” reform, each encompassing various specific measures (sub-themes).

Finally, to identify the strengths and potential gaps in current policies, we quantify the distribution of the policy documents by linkage modes and the WHO six building blocks. All data were analyzed using NVivo 12 (QSR International) software.

## Supplementary information

Supplementary Information

## Data Availability

The study data and related documents are available upon reasonable request from the corresponding author, M.T., via email at maoyi.tian@hrbmu.edu.cn.

## Code availability

Codes to analyze contents of policy documents are available from the Supplementary Information.

## References

[1] Yip, W. et al. 10 years of health-care reform in China: progress and gaps in Universal Health Coverage. *Lancet* **394**, 1192–1204 (2019). 31571602 10.1016/S0140-6736(19)32136-1

[2] Communist Party China Cent. Comm., State Counc. Guidelines for deepening the reform of health care system. https://www.gov.cn/gongbao/content/2009/content_1284372.html (2009).

[3] Liu, G. G., Vortherms, S. A. & Hong, X. China’s health reform update. *Annu. Rev. Public Health* **38**, 431–448 (2017). 28125384 10.1146/annurev-publhealth-031816-044247

[4] National Healthcare Security Administration. Statistical bulletin on healthcare security 2018. http://www.nhsa.gov.cn/art/2019/2/28/art_7_942.html (2019).

[5] Yip, W. C. et al. Early appraisal of China’s huge and complex health-care reforms. *Lancet* **379**, 833–842 (2012). 22386036 10.1016/S0140-6736(11)61880-1

[6] Yip, W. & Hsiao, W. Harnessing the privatisation of China’s fragmented health-care delivery. *Lancet* **384**, 805–818 (2014). 25176551 10.1016/S0140-6736(14)61120-X PMC7159287

[7] Yip, W. C., Hsiao, W., Meng, Q., Chen, W. & Sun, X. Realignment of incentives for health-care providers in China. *Lancet* **375**, 1120–1130 (2010). 20346818 10.1016/S0140-6736(10)60063-3

[8] State Council. Notice on the issuance of Key tasks for Deepening the medical and health System reform in 2014. https://www.gov.cn/zhengce/content/2014-05/28/content_8832.htm (2014).

[9] Wang, L., Wang, Z., Ma, Q., Fang, G. & Yang, J. The development and reform of public health in China from 1949 to 2019. *Glob. Health* **15**, 45 (2019). 10.1186/s12992-019-0486-6 PMC6604346 31266514

[10] Mo, H. et al. Policy idea on breakthrough of health reform in China. Hospital Management in China, 32–35 (2002).

[11] Tu, W. J., Zhong, S. F., Liu, Y. K., Zhan, J. & Liu, Q. The sanming three-in-one model: a potentially useful model for China’s systemic healthcare reform. *J. Am. Geriatr. Soc.* **67**, 2213–2215 (2019). 31355421 10.1111/jgs.16100

[12] State Council. Outline of Healthy China 2030. https://www.gov.cn/zhengce/2016-10/25/content_5124174.htm (2016).

[13] Rao, K. The health reform of the linkage of medical-insurance-medicine and international experience. *Health Econ. Res.* **36**, 4–9 (2019).

[14] Li, L. & Fu, H. China’s health care system reform: progress and prospects. *Int. J. Health Plann. Manag.* **32**, 240–253 (2017). 10.1002/hpm.2424 28612498

[15] Xiao, Y., Husain, L. & Bloom, G. Evaluation and learning in complex, rapidly changing health systems: China’s management of health sector reform. *Glob. Health* **14**, 112 (2018). 10.1186/s12992-018-0429-7 PMC6245843 30454037

[16] Kuruvilla, S. et al. Business not as usual: how multisectoral collaboration can promote transformative change for health and sustainable development. *BMJ* **363**, k4771 (2018). 30530519 10.1136/bmj.k4771 PMC6282730

[17] Fu, H., Li, L., Li, M., Yang, C. & Hsiao, W. An evaluation of systemic reforms of public hospitals: the Sanming model in China. *Health Policy Plan.* **32**, 1135–1145 (2017). 28531288 10.1093/heapol/czx058

[18] Chen, G., Liu, G. G. & Xu, F. The impact of the urban resident basic medical insurance on health services utilisation in China. *Pharmacoeconomics* **32**, 277–292 (2014). 24178373 10.1007/s40273-013-0097-7

[19] Wagstaff, A., Lindelow, M., Jun, G., Ling, X. & Juncheng, Q. Extending health insurance to the rural population: an impact evaluation of China’s new cooperative medical scheme. *J. Health Econ.* **28**, 1–19 (2009). 19058865 10.1016/j.jhealeco.2008.10.007

[20] Zhou, Z. et al. The effect of urban basic medical insurance on health service utilisation in Shaanxi Province, China: a comparison of two schemes. *PLoS ONE* **9**, e94909 (2014). 24740282 10.1371/journal.pone.0094909 PMC3989255

[21] Wang, H. et al. Health insurance benefit design and healthcare utilization in northern rural China. *PLoS ONE* **7**, e50395 (2012). 23185616 10.1371/journal.pone.0050395 PMC3503891

[22] Hu, Y. & Zhang, Z. Skilled doctors in tertiary hospitals are already overworked in China. *Lancet Glob. Health* **3**, e737 (2015). 26566744 10.1016/S2214-109X(15)00192-8

[23] Li, X. et al. The primary health-care system in China. *Lancet* **390**, 2584–2594 (2017). 29231837 10.1016/S0140-6736(17)33109-4

[24] He, Y. et al. Does the leading pharmaceutical reform in China really solve the issue of overly expensive healthcare services? Evidence from an empirical study. *PLoS ONE* **13**, e0190320 (2018). 29338038 10.1371/journal.pone.0190320 PMC5770029

[25] State Council. Circular on policies and measures for further deepening the reform of the medical and health system through the breakthrough of centralized drug procurement and use. https://www.gov.cn/xinwen/2019-12/03/content_5457859.htm (2019).

[26] Chen, L. et al. The impacts of national centralized drug procurement policy on Drug utilization and drug expenditures: the case of Shenzhen, China. *Int. J. Environ. Res. Public Health* **17**, 9415 (2020). 33334027 10.3390/ijerph17249415 PMC7765443

[27] Zhang, X. et al. The impacts and unintended consequences of the nationwide pricing reform for drugs and medical services in the urban public hospitals in China. *BMC Health Serv. Res.* **20**, 1058 (2020). 33225941 10.1186/s12913-020-05849-4 PMC7682084

[28] Jiang, B., Zhou, R. J. & Feng, X. L. The impact of the reference pricing policy in China on drug procurement and cost. *Health Policy Plan.* **37**, 73–99 (2022). 34379765 10.1093/heapol/czab012

[29] Cai, C., Hone, T. & Millett, C. The heterogeneous effects of China’s hierarchical medical system reforms on health service utilisation and health outcomes among elderly populations: a longitudinal quasi-experimental study. *Lancet* **402**, S30 (2023). 37997071 10.1016/S0140-6736(23)02141-4

[30] Zhang, Y., Wang, Q., Jiang, T. & Wang, J. Equity and efficiency of primary health care resource allocation in mainland China. *Int. J. Equity Health* **17**, 140 (2018). 30208890 10.1186/s12939-018-0851-8 PMC6134520

[31] Lai, S. et al. The effects of family physician-contracted service on health-related quality of life and equity in health in China. *Int. J. Equity Health* **20**, 15 (2021). 33407523 10.1186/s12939-020-01348-4 PMC7788691

[32] Zhao, N. & Chen, K. Equity and efficiency of medical and health service system in China. *BMC Health Serv. Res.* **23**, 33 (2023). 36641525 10.1186/s12913-023-09025-2 PMC9840836

[33] State Council. Guidelines on further deepening the payment reform of basic medical insurance. https://www.gov.cn/zhengce/content/2017-06/28/content_5206315.htm (2017).

[34] National Healthcare Security Administration. Guidelines on further deepening the payment reform of basic medical insurance. https://www.gov.cn/zhengce/zhengceku/2021-11/28/content_5653858.htm (2021).

[35] Zhang, Y., Ma, Q., Chen, Y. & Gao, H. Effects of public hospital reform on inpatient expenditures in rural China. *Health Econ.* **26**, 421–430 (2017). 26842555 10.1002/hec.3320

[36] He, R. et al. The effects of global budget on cost control and readmission in rural China: a difference-in-difference analysis. *J. Med. Econ.* **20**, 903–910 (2017). 28562140 10.1080/13696998.2017.1336448

[37] Huang, Y., Liu, Y., Yang, X., Li, J. & Fang, P. Global budget payment system helps to reduce outpatient medical expenditure of hypertension in China. *Springerplus* **5**, 1877 (2016). 27833836 10.1186/s40064-016-3565-7 PMC5081988

[38] Jian, W. et al. Payment reform pilot in Beijing hospitals reduced expenditures and out-of-pocket payments per admission. *Health Aff.* **34**, 1745–1752 (2015). 10.1377/hlthaff.2015.0074 26438752

[39] Li, H. M. et al. Effectiveness evaluation of quota payment for specific diseases under global budget: a typical provider payment system reform in rural China. *BMC Health Serv. Res.* **18**, 635 (2018). 30103736 10.1186/s12913-018-3415-0 PMC6090661

[40] Peng, J., Zhang, M., Yu, P. & Wang, N. Can single disease payment system based on clinical pathway reduce hospitalization costs in rural area? A case study of uterine leiomyoma in Anhui, China. *BMC Health Serv. Res.* **18**, 990 (2018). 30572899 10.1186/s12913-018-3807-1 PMC6302448

[41] He, R. et al. Medical service quality, efficiency and cost control effectiveness of upgraded case payment in rural China: a retrospective study. *Int. J. Environ. Res. Public Health* **15**, 2839 (2018). 30551561 10.3390/ijerph15122839 PMC6313562

[42] Liu, S., Wang, J., Zhang, L. & Zhang, X. Caesarean section rate and cost control effectiveness of case payment reform in the new cooperative medical scheme for delivery: evidence from Xi County, China. *BMC Pregnancy Childbirth* **18**, 66 (2018). 29523121 10.1186/s12884-018-1698-0 PMC5845290

[43] Yang, W. et al. Understanding health and social challenges for aging and long-term care in China. *Res. Aging* **43**, 127–135 (2021). 32677535 10.1177/0164027520938764 PMC7961665

[44] Meng, Q., Mills, A., Wang, L. & Han, Q. What can we learn from China’s health system reform? *BMJ* **365**, l2349 (2019). 31217222 10.1136/bmj.l2349 PMC6598719

[45] Moher, D., Liberati, A., Tetzlaff, J. & Altman, D. G. Preferred reporting items for systematic reviews and meta-analyses: the PRISMA Statement. *Open Med.* **3**, e123–e130 (2009). 21603045 PMC3090117

