# Supplementary material for: A scoping review of Chinese national policies to promote the three-medicals linkage reform since 2014
Source: Npj Health Syst. 2025 Mar 28;2:9. doi: 10.1038/s44401-025-00012-9 (PMC13354206; doi:10.1038/s44401-025-00012-9)
Supplement: Supplementary file 1 — Supplementary Information [file 44401_2025_12_MOESM1_ESM.pdf]

Supplementary Information

Supplementary Table 1: Preferred Reporting Items for Systematic reviews and Meta-Analyses extension for Scoping Reviews (PRISMA-ScR) Checklist

Supplementary Table 2: National Ministry website for policy search

Supplementary Table 3: List of Included National-Level Policy Documents

Supplementary Table 4: The study coding book.

Supplementary Figure 1: Eligible policy documents by three time periods.

Supplementary Figure 2: Distribution of policies containing the WHO's six building blocks of health system over three time periods

Supplementary Table 1: Preferred Reporting Items for Systematic reviews and Meta-Analyses extension for Scoping Reviews (PRISMA-ScR) Checklist

SECTION	ITEM	PRISMA-ScR CHECKLIST ITEM	REPORTED ON PAGE #
**TITLE**			
Title	1	Identify the report as a scoping review.	Page 1
**ABSTRACT**			
Structured summary	2	Provide a structured summary that includes (as applicable): background, objectives, eligibility criteria, sources of evidence, charting methods, results, and conclusions that relate to the review questions and objectives.	Page 2
**INTRODUCTION**			
Rationale	3	Describe the rationale for the review in the context of what is already known. Explain why the review questions/objectives lend themselves to a scoping review approach.	Page 3
Objectives	4	Provide an explicit statement of the questions and objectives being addressed with reference to their key elements (e.g., population or participants, concepts, and context) or other relevant key elements used to conceptualize the review questions and/or objectives.	Page 3
**METHODS**			
Protocol and registration	5	Indicate whether a review protocol exists; state if and where it can be accessed (e.g., a Web address); and if available, provide registration information, including the registration number.	Page 4
Eligibility criteria	6	Specify characteristics of the sources of evidence used as eligibility criteria (e.g., years considered, language, and publication status), and provide a rationale.	Page 6
Information sources*	7	Describe all information sources in the search (e.g., databases with dates of coverage and contact with authors to identify additional sources), as well as the date the most recent search was executed.	Page 6
Search	8	Present the full electronic search strategy for at least 1 database, including any limits used, such that it could be repeated.	Page 6
Selection of sources of evidence†	9	State the process for selecting sources of evidence (i.e., screening and eligibility) included in the scoping review.	Page 6
Data charting process‡	10	Describe the methods of charting data from the included sources of evidence (e.g., calibrated forms or forms that have been tested by the team before their use, and whether data charting was done independently or in duplicate) and any processes for obtaining and confirming data from investigators.	Page 6, 7
Data items	11	List and define all variables for which data were sought and any assumptions and simplifications made.	Page 6, 7
Critical appraisal of individual sources of evidence§	12	If done, provide a rationale for conducting a critical appraisal of included sources of evidence; describe the methods used and how this information was used in any data synthesis (if appropriate).	NA
Synthesis of results	13	Describe the methods of handling and summarizing the data that were charted.	Page 7
**RESULTS**			

SECTION	ITEM	PRISMA-ScR CHECKLIST ITEM	REPORTED ON PAGE #
Selection of sources of evidence	14	Give numbers of sources of evidence screened, assessed for eligibility, and included in the review, with reasons for exclusions at each stage, ideally using a flow diagram.	Page 8, 9
Characteristics of sources of evidence	15	For each source of evidence, present characteristics for which data were charted and provide the citations.	Page 8-18
Critical appraisal within sources of evidence	16	If done, present data on critical appraisal of included sources of evidence (see item 12).	NA
Results of individual sources of evidence	17	For each included source of evidence, present the relevant data that were charted that relate to the review questions and objectives.	Appendix 3
Synthesis of results	18	Summarize and/or present the charting results as they relate to the review questions and objectives.	Page 8-18
**DISCUSSION**			
Summary of evidence	19	Summarize the main results (including an overview of concepts, themes, and types of evidence available), link to the review questions and objectives, and consider the relevance to key groups.	Page 19
Limitations	20	Discuss the limitations of the scoping review process.	Page 21
Conclusions	21	Provide a general interpretation of the results with respect to the review questions and objectives, as well as potential implications and/or next steps.	Page 22
**FUNDING**			
Funding	22	Describe sources of funding for the included sources of evidence, as well as sources of funding for the scoping review. Describe the role of the funders of the scoping review.	Page 23

JBI = Joanna Briggs Institute; PRISMA-ScR = Preferred Reporting Items for Systematic reviews and Meta-Analyses extension for Scoping Reviews.

* Where *sources of evidence* (see second footnote) are compiled from, such as bibliographic databases, social media platforms, and Web sites.

† A more inclusive/heterogeneous term used to account for the different types of evidence or data sources (e.g., quantitative and/or qualitative research, expert opinion, and policy documents) that may be eligible in a scoping review as opposed to only studies. This is not to be confused with *information sources* (see first footnote).

‡ The frameworks by Arksey and O'Malley (6) and Levac and colleagues (7) and the JBI guidance (4, 5) refer to the process of data extraction in a scoping review as data charting.

§ The process of systematically examining research evidence to assess its validity, results, and relevance before using it to inform a decision. This term is used for items 12 and 19 instead of "risk of bias" (which is more applicable to systematic reviews of interventions) to include and acknowledge the various sources of evidence that may be used in a scoping review (e.g., quantitative and/or qualitative research, expert opinion, and policy document).

From: Tricco AC, Lillie E, Zarin W, O'Brien KK, Colquhoun H, Levac D, et al. PRISMA Extension for Scoping Reviews (PRISMAScR): Checklist and Explanation. Ann Intern Med. 2018;169:467–473. doi: 10.7326/M18-0850.

Supplementary Table 2: National Ministry website for policy search

Serial number	Name of Ministry in Chinese	Name of Ministry in Englishi	Ministry Websites
1	国家卫生健康委员会	National Health Commission	http://www.nhc.gov.cn/
2	人力资源和社会保障部	Ministry of Human Resources and Social Security	http://www.mohrss.gov.cn/
3	财政部	Ministry of Finance	http://www.mof.gov.cn/index.htm
4	国家药品监督管理局	State Drug Administration	http://www.nmpa.gov.cn/WS04/CL2042/
5	国家中医药管理局	National Administration of Traditional Chinese Medicine	http://www.satcm.gov.cn/
6	国家医疗保障局	National Healthcare Security Administration	http://www.nhsa.gov.cn/
7	科学技术部	Ministry of Science and Technology	http://www.most.gov.cn/
8	国家发展和改革委员会	National Development and Reform Commission	http://www.ndrc.gov.cn/
9	民政部	Ministry of Civil Affairs	http://www.mca.gov.cn/
10	工业和信息化部	Ministry of Industry and Information Technology	http://www.miit.gov.cn/
11	住房和城乡建设部	Ministry of Housing and Urban-Rural Development	http://www.mohurd.gov.cn/
12	生态环境部	Ministry of Ecology and Environment	http://www.mee.gov.cn/
13	教育部	Ministry of Education	http://www.moe.gov.cn/
14	农业农村部	Ministry of Agriculture and Rural Affairs	http://www.moa.gov.cn/
15	国家体育总局	General Administration of Sport of China	http://www.sport.gov.cn/
16	国家机关事务管理局	National Government Offices Administration	http://www.ggj.gov.cn/
17	国务院发展研究中心	Development Research Center of The State Council	http://www.drc.gov.cn/
18	中国科学院	Chinese Academy of Sciences	http://www.cas.cn/
19	中国社会科学院	Chinese Academy of Social Sciences	http://cass.cssn.cn/
20	国家统计局	National Bureau of Statistics	http://www.stats.gov.cn

Supplementary Table 3: List of Included National-Level Policy Documents

Serial number	Chinese title	English title	Time of release	Releasing departments	Referenced documents (by serial number)
	12th five-year plan (2014-2015）				
1	关于印发深化医药卫生体制改革 2014 年重点工作任务的通知	Notice on the issuance of Key tasks for Deepening the medical and health System reform in 2014	28-05-2014	The State Council	
2	关于做好常用低价药品采购管理工作的通知	Notice on the Procurement Management of Commonly Used low-cost Drugs	04-06-2014	National Health Commission	
3	关于抓好 2014 年县级公立医院综合改革试点工作落实的通知	Notice on the implementation of the pilot reform of county-level public hospitals in 2014	27-06-2014	National Health Commission	
4	关于加快发展商业健康保险的若干意见	Some Opinions on Accelerating the Development of Commercial Health Insurance	17-11-2014	The State Council	
5	关于印发 2015 年卫生计生工作要点的通知	Notice on the Issuance of Key Points of Health and Family Planning Work in 2015	23-01-2015	National Health Commission	
6	关于做好 2015 年新型农村合作医疗工作的通知	Notice on improving the work of the new rural Cooperative medical System in 2015	29-01-2015	National Health Commission	
7	关于完善公立医院药品集中采购工作的指导意见	Guiding Opinions on improving the centralized procurement of drugs in public hospitals	28-02-2015	The State Council	
8	关于印发全国医疗卫生服务体系规划纲要（2015—2020 年）的通知	Notice on Issuing the Outline of the Plan for the National Medical and Health Service System (2015-2020)	30-03-2015	The State Council	
9	关于落实《政府工作报告》重点工作部门分工的意见 2015	Opinions on the Implementation of the Division of Labor of Key Work Departments in the Government Work Report 2015	10-04-2015	The State Council	

Continue

10	关于印发推进药品价格改革意见的通知	Notice on Issuing Opinions on Advancing Drug Price Reform	07-05-2015	National Health Commission	
11	关于全面推开县级公立医院综合改革的实施意见	Implementation Opinions on comprehensively promoting the comprehensive reform of county-level public hospitals	08-05-2015	The State Council	
12	关于印发深化医药卫生体制改革 2014 年工作总结和 2015 年重点工作任务的通知	Notice on the issuance of the Summary of the work in 2014 and the key tasks in 2015 for deepening the medical and health system Reform	09-05-2015	The State Council	
13	关于城市公立医院综合改革试点的指导意见	Guiding opinions on the pilot reform of Urban public hospitals	17-05-2015	The State Council	
14	关于 2015 年深化经济体制改革重点工作意见的通知	Notice on the Opinions on the Key Work of Deepening Economic Restructuring in 2015	18-05-2015	The State Council	9
15	关于促进社会办医加快发展若干政策措施的通知	Notice on Policies and Measures to Accelerate the Development of Private hospitals	15-06-2015	The State Council	
16	关于落实完善公立医院药品集中采购工作指导意见的通知	Notice of the National Health and Family Planning Commission on implementing and improving the work of centralized drug procurement at public Hospitals	19-06-2015	National Health Commission	7
17	关于全面实施城乡居民大病保险的意见	Opinions on the Full Implementation of Serious Illness Insurance for Urban and Rural Residents	02-08-2015	The State Council	
18	关于推进分级诊疗制度建设的指导意见	Guiding opinions on promoting the construction of hierarchical medical system	11-09-2015	The State Council	
19	关于深入开展创建“平安医院”活动依法维护医疗秩序的意见	Opinions on deepening the establishment of "safe hospital" activities to maintain medical order according to law	09-10-2015	National Health Commission	
20	关于印发控制公立医院医疗费用不合理增长的若干意见的通知	Notice on the Issuance of Certain Opinions on Controlling Unreasonable Growth of Medical Expenses in Public Hospitals	06-11-2015	National Health Commission	
21	关于进一步规范社区卫生服务管理和提升服务质量的指导意见	Guiding opinions on further standardizing community health service management and improving service quality	25-11-2015	National Health Commission	18

Continue

	**13th five-year plan (2016-2020）**				
22	关于整合城乡居民基本医疗保险制度的意见	Opinions on the Integration of the Basic Medical Insurance System for Urban and Rural Residents	12-01-2016	The State Council	
23	关于做好贯彻落实《国务院关于整合城乡居民基本医疗保险制度的意见》有关工作的通知	Notice on the Work Related to the Implementation of the Opinions of The State Council on the Integration of the Basic Medical Insurance System for Urban and Rural Residents	13-01-2016	Ministry of Human Resources and Social Security	22
24	关于做好整合城乡居民基本医疗保险制度有关工作的通知	Notice on the Work of Integrating the Basic Medical Insurance System for Urban and Rural Residents	21-01-2016	National Health Commission	22
25	关于促进医药产业健康发展的指导意见	Guiding Opinions on Promoting the Healthy Development of Pharmaceutical Industry	11-03-2016	The State Council	
26	关于落实《政府工作报告》重点工作部门分工的意见 2016	Opinions on the Implementation of the Division of Labor of Key Work Departments in the Government Work Report 2016	29-03-2016	The State Council	
27	关于 2016 年深化经济体制改革重点工作意见的通知	Notice on the Opinions on the Key Work of Deepening Economic Restructuring in 2016	31-03-2016	The State Council	26
28	关于印发深化医药卫生体制改革 2016 年重点工作任务的通知	Notice on Issuing Key tasks for Deepening the reform of the medical and health System in 2016	26-04-2016	The State Council	
29	关于做好 2016 年城镇居民基本医疗保险工作的通知	Notice on the Work of Basic Medical Insurance for Urban Residents in 2016	29-04-2016	Ministry of Human Resources and Social Security	
30	关于做好 2016 年新型农村合作医疗工作的通知	Notice on improving the work of the New rural Cooperative medical System in 2016	05-05-2016	National Health Commission	22
31	关于印发加强儿童医疗卫生服务改革与发展意见的通知	Notice on Issuing Opinions on Strengthening the Reform and Development of Children's Medical and Health Services	18-05-2016	National Health Commission	8
32	关于做好国家谈判药品集中采购的通知	Notice on Centralized Procurement of Drugs through national Negotiations	20-05-2016	National Health Commission	

Continue

33	关于印发推进家庭医生签约服务指导意见的通知	The sign about print and distribute to promote family doctor service guidance notice	06-06-2016	The State Council	18
34	关于促进和规范健康医疗大数据应用发展的指导意见	Guiding opinions on promoting and regulating the development of big data applications in healthcare	24-06-2016	The State Council	
35	关于积极推动医疗、医保、医药联动改革的指导意见	Guiding Opinions on actively promoting the linkage reform of medical care, medical insurance and medicine	29-06-2016	Ministry of Human Resources and Social Security	
36	关于推进分级诊疗试点工作的通知	Notice on promoting the pilot work of hierarchical medical system	19-08-2016	National Health Commission	18, 28
37	关于做好 2016 年县级公立医院综合改革工作的通知	Regarding the 2016 public hospitals at the county level comprehensive reform work notice	18-09-2016	National Health Commission	11, 28
38	“健康中国 2030”规划纲要	Outline of Healthy China 2030	25-10-2016	The State Council	
39	关于进一步推广深化医药卫生体制改革经验的若干意见	Opinions on further promoting and deepening the experience of the medical and health system reform	08-11-2016	The State Council	
40	关于印发“十三五”脱贫攻坚规划的通知	Notice on the Issuance of the 13th Five-Year Plan for Poverty Alleviation	02-12-2016	The State Council	
41	关于加强基本医疗保险基金预算管理发挥医疗保险基金控费作用的意见	Opinions on strengthening the budget management of basic medical insurance funds and giving full play to the role of medical insurance funds in controlling costs	29-12-2016	Ministry of Finance	
42	关于印发“十三五”深化医药卫生体制改革规划的通知	Notice on the Issuance of the 13th Five-Year Plan for Deepening the Reform of the medical and Health System	09-01-2017	The State Council	38
43	关于印发“十三五”卫生与健康规划的通知	Notice on the Issuance of the 13th Five-Year Plan on Health and Wellness	10-01-2017	The State Council	38
44	关于开展公立医院薪酬制度改革试点工作的指导意见	Guiding Opinions on the pilot work of the salary system reform in public hospitals	24-01-2017	Ministry of Human Resources and Social Security	

Continue

45	关于进一步改革完善药品生产流通使用政策的若干意见	Several Opinions on Further Reforming and Improving the Policy of Drug Production, Circulation and Use	09-02-2017	The State Council	
46	关于印发中国防治慢性病中长期规划（2017—2025 年）的通知	Notice on Issuing China's Medium - and long-term Plan for the Prevention and Control of Non-communicable Diseases (2017-2025)	14-02-2017	The State Council	
47	关于印发“十三五”全国人口健康信息化发展规划的通知	Notice on the Issuance of the 13th Five-Year National Population Health Informatization Development Plan	21-02-2017	National Health Commission	38, 34, 43
48	关于印发农村贫困人口大病专项救治工作方案的通知	Notice on the Issuance of the Work Plan for the Special Treatment of Serious Diseases for the Rural Poor	23-02-2017	National Health Commission	
49	关于印发“十三五”推进基本公共服务均等化规划的通知	Notice on the Issuance of the 13th Five-Year Plan for Promoting the Equalization of Basic Public Services	01-03-2017	The State Council	
50	关于 2017 年深化经济体制改革重点工作意见的通知	Notice on the Opinions on the Key Work of Deepening Economic Restructuring in 2017	18-04-2017	The State Council	
51	关于做好 2017 年新型农村合作医疗工作的通知	Notice on improving the work of the New rural Cooperative medical System in 2017	20-04-2017	National Health Commission	
52	关于全面推开公立医院综合改革工作的通知	Notice on Comprehensively promoting the comprehensive reform of Public Hospitals	24-04-2017	National Health Commission	42
53	关于做好 2017 年城镇居民基本医疗保险工作的通知	Notice on the Work of Basic Medical Insurance for Urban Residents in 2017	24-04-2017	Ministry of Human Resources and Social Security	
54	关于推进医疗联合体建设和发展的指导意见	Guiding Opinions on promoting the construction and development of medical alliances	26-04-2017	The State Council	
55	关于印发深化医药卫生体制改革 2017 年重点工作任务的通知	Notice on issuing Key tasks for Deepening the medical and health System Reform in 2017	05-05-2017	The State Council	

Continue

56	关于印发《"十三五"健康产业科技创新专项规划》的通知	Notice on the Issuance of the 13th Five-Year Plan for Scientific and Technological Innovation in the Health Industry	14-06-2017	Ministry of Science and Technology	38
57	关于进一步深化基本医疗保险支付方式改革的指导意见	Guidance on further deepening the reform of basic medical insurance Payment Methods	28-06-2017	The State Council	
58	关于改革完善短缺药品供应保障机制的实施意见	Implementation Opinions on Reforming and improving the Supply guarantee Mechanism of Drugs in Shortage	28-06-2017	National Health Commission	38, 42, 43, 45
59	关于做好国家卫生计生委和国家中医药局属管医院参加属地公立医院综合改革有关工作的通知	Notice on the Participation of hospitals under the National Health and Family Planning Commission and the State Administration of Traditional Chinese Medicine in the comprehensive reform of territorial public hospitals	04-07-2017	National Health Commission	55
60	关于印发进一步改善医疗服务行动计划（2018-2020 年）的通知	Notice on Issuing the Action Plan for Further Healthcare Improvement (2018-2020)	04-01-2018	National Health Commission	
61	关于改革完善全科医生培养与使用激励机制的意见	Suggestions on reforming and improving the incentive mechanism for training and using general practitioners	14-01-2018	The State Council	38
62	关于巩固破除以药补医成果持续深化公立医院综合改革的通知	Notice on Consolidating the achievements of drug supplement medicine and continuously deepening the comprehensive reform of public hospitals	20-03-2018	National Health Commission	
63	关于做好 2018 年家庭医生签约服务工作的通知	Notice on family Doctor Contracted Service in 2018	02-04-2018	National Health Commission	
64	关于开展抗癌药省级专项集中采购工作的通知	Notice on Carrying out the work of provincial Special Centralized Procurement of Anticancer Drugs	17-07-2018	National Healthcare Security Administration	
65	关于改革完善医疗卫生行业综合监管制度的指导意见	Guiding Opinions on Reforming and improving the Comprehensive supervision System of the medical and health Industry	03-08-2018	The State Council	

Continue

66	关于进一步做好分级诊疗制度建设有关重点工作的通知	Notice on further key work in the construction of hierarchical medical system	10-08-2018	National Health Commission	18, 54
67	关于印发医疗卫生领域中央与地方财政事权和支出责任划分改革方案的通知	Notice on the Issuance of the Reform Plan on the Division of financial Powers and expenditure Responsibilities between the Central and local Governments in the medical and health Field	13-08-2018	The State Council	
68	关于坚持以人民健康为中心推动医疗服务高质量发展的意见	Opinions on Promoting the high-quality development of medical services centering on people's health	16-08-2018	National Health Commission	
69	关于进一步加强医务人员队伍建设的通知	Notice on further strengthening the construction of medical staff	27-08-2018	National Health Commission	
70	关于印发深化医药卫生体制改革 2018 年下半年重点工作任务的通知	Notice on the issuance of Key tasks for Deepening the medical and health System reform in the second half of 2018	28-08-2018	The State Council	
71	关于完善国家基本药物制度的意见	Opinions on improving the National Essential Medicine System	19-09-2018	The State Council	
72	关于印发全面提升县级医院综合能力工作方案（2018-2020 年）的通知	Notice on Issuing the Work Plan for Comprehensively Improving the Comprehensive Capacity of county-level hospitals (2018-2020	08-11-2018	National Health Commission	11
73	关于加快推进电子健康卡普及应用工作的意见	Opinions on accelerating the popularization and application of the electronic health Card	21-12-2018	National Health Commission	
74	关于印发加快落实仿制药供应保障及使用政策工作方案的通知	Notice on Printing and Issuing the Work Plan for Accelerating the Implementation of the Policy on Supply Security and Use of Generic Drugs	29-12-2018	National Health Commission	
75	关于印发国家组织药品集中采购和使用试点方案的通知	Notice on Issuing the Pilot Plan for Centralized Procurement and Use of Drugs Organized by the State	17-01-2019	The State Council	

Continue

76	关于落实《政府工作报告》重点工作部门分工的意见 2019	Opinions on the Implementation of the Division of Labor of Key Work Departments in the Government Work Report 2019	09-04-2019	The State Council	
77	关于开展药品使用监测和临床综合评价工作的通知	Notice on the Implementation of Drug use Monitoring and Clinical Comprehensive Evaluation	09-04-2019	National Health Commission	38, 42, 43
78	关于印发深化医药卫生体制改革 2019 年重点工作任务的通知	Notice on issuing key tasks for Deepening the medical and health system reform in 2019	04-06-2019	The State Council	
79	关于印发促进社会办医持续健康规范发展意见的通知	Notice on issuing Opinions on Promoting the Sustainable and Healthy Development of Private hospitals	12-06-2019	National Health Commission	
80	关于印发治理高值医用耗材改革方案的通知	Notice on the Printing and Distribution of the reform Plan for the management of high-value medical consumables	31-07-2019	The State Council	
81	关于国家组织药品集中采购和使用试点扩大区域范围的实施意见	Implementation Opinions on expanding the Regional scope of Trials for Centralized Drug Procurement and Use organized by the State	30-09-2019	National Healthcare Security Administration	75
82	关于进一步做好短缺药品保供稳价工作的意见	Opinions on Further ensuring supply and price stability of drugs in Shortage	11-10-2019	The State Council	
83	关于促进中医药传承创新发展的意见	Opinions on Promoting the Inheritance, Innovation and Development of Traditional Chinese Medicine	20-10-2019	The State Council	
84	关于进一步推广福建省和三明市深化医药卫生体制改革经验的通知	Notice on further promoting the experience of Fujian Province and Sanming City in deepening the reform of the medical and health system	15-11-2019	The State Council	
85	关于以药品集中采购和使用为突破口进一步深化医药卫生体制改革若干政策措施的通知	Notice on Policies and Measures for Further Deepening the reform of the medical and health System through the Breakthrough of Centralized Drug Procurement and Use	29-11-2019	The State Council	
86	关于做好当前药品价格管理工作的意见	Opinions on the current Management of Drug Prices	06-12-2019	National Healthcare Security Administration	

Continue

87	关于做好 2019 年国家医保谈判药品落地工作的通知	Notice on the landing of drugs negotiated by the national Medical Insurance in 2019	18-12-2019	National Healthcare Security Administration	
88	关于开展第二批国家组织药品集中采购和使用工作的通知	Notice on Carrying out the second batch of State-organized Centralized procurement and Use of Drugs	16-01-2020	National Healthcare Security Administration	75, 81
89	关于深化医疗保障制度改革的意见	Opinions on Deepening the Reform of the medical security System	25-02-2020	The State Council	
90	关于开展国家组织药品集中采购和使用中选药品专项检查工作的通知	Notice on Carrying out the work of Special Inspection on the Centralized Procurement and Use of Selected Drugs organized by the State	10-03-2020	State Drug Administration	
91	关于推进医疗保障基金监管制度体系改革的指导意见	Guiding Opinions on promoting the Reform of the supervision system of medical security Fund	09-07-2020	The State Council	
92	关于印发深化医药卫生体制改革 2020 年下半年重点工作任务的通知	Notice on Issuing Key tasks for Deepening the medical and health System Reform in the second half of 2020	23-07-2020	The State Council	
93	基本医疗保险用药管理暂行办法	Interim Measures for Drug Administration under Basic Medical Insurance	30-07-2020	National Healthcare Security Administration	89
94	关于制定国民经济和社会发展第十四个五年规划和二〇三五年远景目标的建议	Suggestions on the Formulation of the 14th Five-Year Plan for National Economic and Social Development and the Vision for 2035	29-10-2020	The State Council	
95	关于深入推进“互联网+医疗健康”“五个一”服务行动的通知	Notice on in-depth Promotion of "Internet + Healthcare" and "Five Ones" Service Actions	15-12-2020	National Health Commission	

Continue

	**Thirteenth five-year plan (2016-2020）**			
96	关于推动药品集中带量采购工作常态化制度化开展的意见	Opinions on promoting the normalization and institutionalization of centralized drug procurement in large quantities	28-01-2021	The State Council
97	关于落实《政府工作报告》重点工作部门分工的意见 2021	Opinions on the Implementation of the Division of Labor of Key Work Departments in the Government Work Report 2021	25-03-2021	The State Council
98	关于建立健全职工基本医疗保险门诊共济保障机制的指导意见	Guiding Opinions on establishing and improving the Mechanism for Outpatient Mutual assistance under employee basic medical Insurance	22-04-2021	The State Council
99	关于开展国家组织高值医用耗材集中带量采购和使用的指导意见	Guiding opinions on carrying out state-organized centralized procurement and use of high-value medical consumables in large quantities	04-06-2021	National Healthcare Security Administration
100	关于推动公立医院高质量发展的意见	Opinions on promoting the high-quality development of public hospitals	04-06-2021	The State Council
101	关于印发深化医药卫生体制改革 2021 年重点工作任务的通知	Notice on issuing key tasks for Deepening the medical and health system reform in 2021	17-06-2021	The State Council
102	关于深化公立医院薪酬制度改革的指导意见	Guiding opinions on deepening the reform of the salary system of public hospitals	06-07-2021	Ministry of Human Resources and Social Security
103	深化医疗服务价格改革试点方案	Pilot plan for Deepening medical service price Reform	31-08-2021	National Healthcare Security Administration
104	关于印发中国妇女发展纲要和中国儿童发展纲要的通知	Notice on the Issuance of the Outline for the Development of Chinese Women and the Outline for the Development of Chinese Children	27-09-2021	The State Council
105	关于印发“十四五”全民医疗保障规划的通知	Notice on the Issuance of the 14th Five-Year Plan for Universal Medical Security	29-09-2021	The State Council

Continue

106	关于深入推广福建省三明市经验 深化医药卫生体制改革的实施意见	Implementation opinions on deepening the medical and health system reform based on the experience of Sanming City, Fujian Province	08-10-2021	The State Council	
107	关于印发 DRG/DIP 支付方式改革三年行动计划的通知	Notice on Issuing the three-year Action Plan for DRG/DIP Payment Reform	26-11-2021	National Healthcare Security Administration	89
108	关于抓好深入推广福建省三明市经验 深化医药卫生体制改革实施意见落实的通知	Notice on Promoting the experience of Sanming City in Fujian Province and deepening the implementation of the implementation opinions on the medical and health system reform	29-12-2021	The State Council	
109	"十四五"国家药品安全及促进高质量发展规划	14th Five-Year Plan for Drug Safety and High-quality Development	30-12-2021	State Drug Administration	94
110	关于做好 2022 年全面推进乡村振兴重点工作的意见	Opinions on comprehensively promoting the key Work of Rural Revitalization in 2022	23-02-2022	The State Council	
111	关于印发"十四五"中医药发展规划的通知	Notice on the Issuance of the 14th Five-Year Plan for the Development of Traditional Chinese Medicine	29-03-2022	The State Council	
112	关于印发"十四五"国民健康规划的通知	Notice on the Issuance of the 14th Five-Year National Health Plan	20-05-2022	The State Council	
113	关于印发深化医药卫生体制改革 2022 年重点工作任务的通知	Notice on issuing key tasks for deepening the medical and health system reform in 2022	25-05-2022	The State Council	
114	关于做好 2022 年城乡居民基本医疗保障工作的通知	Notice on Ensuring Basic medical Security for Urban and Rural Residents in 2022	08-07-2022	National Healthcare Security Administration	
115	关于进一步做好基本医疗保险跨省异地就医直接结算工作的通知	Notice on Further Improving the Cross-provincial Direct Settlement of Basic Medical Insurance	26-07-2022	National Healthcare Security Administration	89
116	关于进一步深化改革促进乡村医疗卫生体系健康发展的意见	Opinions on further deepening reform to promote the Healthy Development of Rural medical and health System	23-02-2023	The State Council	

Continue

117	关于印发中医药振兴发展重大工程实施方案的通知	Notice on the Issuance of the Implementation Plan of the Major Project for the Revitalization and Development of Traditional Chinese Medicine	28-02-2023	The State Council
118	关于进一步完善医疗卫生服务体系的意见	Opinions on further improving the medical and health service System	23-03-2023	The State Council
119	关于加强医疗保障基金使用常态化监管的实施意见	Implementation Opinions on strengthening the regular supervision of the use of medical security funds	26-05-2023	The State Council
120	关于印发深化医药卫生体制改革 2023 年下半年重点工作任务的通知	Notice on Issuing key tasks for Deepening the medical and health system reform in the second half of 2023	21-07-2023	The State Council
121	关于全面推进紧密型县域医疗卫生共同体建设的指导意见	Guiding Opinions on comprehensively promoting the construction of compact county-wide medical and health Communities	30-12-2023	National Health Commission

Supplementary Table 4: The study coding book.

Themes	Number of policy documents	Number of codes
**WHO's six building blocks of health system**	121	1142
**Leadership and governance**	78	228
Medical care and medical insurance linkage mode	43	84
Medical care and medicine linkage mode	37	61
Medical care, medicine and medical insurance linkage mode	32	47
Medicine and medical insurance linkage mode	17	30
**Service delivery**	86	252
Medical care and medical insurance linkage mode	60	140
Medical care and medicine linkage mode	43	64
Medical care, medicine and medical insurance linkage mode	26	30
Medicine and medical insurance linkage mode	0	0
**Health workforce**	21	28
Medical care and medical insurance linkage mode	6	10
Medical care and medicine linkage mode	14	15
Medical care, medicine and medical insurance linkage mode	2	2
Medicine and medical insurance linkage mode	1	1
**Health information**	52	87
Medical care and medical insurance linkage mode	23	29
Medical care and medicine linkage mode	15	17
Medical care, medicine and medical insurance linkage mode	16	20
Medicine and medical insurance linkage mode	13	15
**Health financing**	95	348
Medical care and medical insurance linkage mode	74	173

Medical care and medicine linkage mode	27	47
Medical care, medicine and medical insurance linkage mode	33	49
Medicine and medical insurance linkage mode	37	62
**Medicines and technologies**	72	199
Medical care and medical insurance linkage mode	0	0
Medical care and medicine linkage mode	45	97
Medical care, medicine and medical insurance linkage mode	25	26
Medicine and medical insurance linkage mode	39	64
**12 major policy strategies**	121	669
**Hierarchical medical system**	29	36
Medical care and medical insurance linkage mode	23	29
Medical care and medicine linkage mode	6	6
Medical care, medicine and medical insurance linkage mode	1	1
Medicine and medical insurance linkage mode	0	0
**Support private medical institutions**	6	7
Medical care and medical insurance linkage mode	6	7
Medical care and medicine linkage mode	0	0
Medical care, medicine and medical insurance linkage mode	0	0
Medicine and medical insurance linkage mode	0	0
**Personnel and salary system of public medical institutions**	28	47
Medical care and medical insurance linkage mode	12	14
Medical care and medicine linkage mode	15	22
Medical care, medicine and medical insurance linkage mode	9	10
Medicine and medical insurance linkage mode	0	0

**Construction of primary healthcare workers**	2	2
Medical care and medical insurance linkage mode	2	2
Medical care and medicine linkage mode	0	0
Medical care, medicine and medical insurance linkage mode	0	0
Medicine and medical insurance linkage mode	0	0
**Reasonable control of medical service expenses**	40	73
Medical care and medical insurance linkage mode	18	26
Medical care and medicine linkage mode	16	24
Medical care, medicine and medical insurance linkage mode	15	18
Medicine and medical insurance linkage mode	5	5
**Reform of medical insurance payment methods**	63	143
Medical care and medical insurance linkage mode	58	111
Medical care and medicine linkage mode	0	0
Medical care, medicine and medical insurance linkage mode	18	24
Medicine and medical insurance linkage mode	8	8
**The essential drug system and drug supply guarantee**	25	45
Medical care and medical insurance linkage mode	0	0
Medical care and medicine linkage mode	12	18
Medical care, medicine and medical insurance linkage mode	2	2
Medicine and medical insurance linkage mode	15	25
**Standardization of medical services**	38	61
Medical care and medical insurance linkage mode	4	5
Medical care and medicine linkage mode	30	44
Medical care, medicine and medical insurance linkage mode	10	10
Medicine and medical insurance linkage mode	2	2

**Construction of medical alliances**	8	8
Medical care and medical insurance linkage mode	5	5
Medical care and medicine linkage mode	2	2
Medical care, medicine and medical insurance linkage mode	1	1
Medicine and medical insurance linkage mode	0	0
**Universal medical insurance and informatization of medical insurance**	46	94
Medical care and medical insurance linkage mode	42	80
Medical care and medicine linkage mode	1	1
Medical care, medicine and medical insurance linkage mode	9	10
Medicine and medical insurance linkage mode	3	3
**Family doctor system**	12	23
Medical care and medical insurance linkage mode	9	12
Medical care and medicine linkage mode	3	8
Medical care, medicine and medical insurance linkage mode	3	3
Medicine and medical insurance linkage mode	0	0
**Centralized drug procurement**	50	130
Medical care and medical insurance linkage mode	3	3
Medical care and medicine linkage mode	26	59
Medical care, medicine and medical insurance linkage mode	11	14
Medicine and medical insurance linkage mode	34	54

	12th Five-Year Plan (2014-2015)	13th Five-Year Plan (2016-2020)	14th Five-Year Plan (2021-2023)	All
**Number of all documents**	21	74	26	121
**Releasing entities**				
State of Council	15	42	15	72
National Health Commission[2]	6	26	4	36
MO Finance	3	14	6	23
MO human resources and social security	2	14	3	19
National Admin of Traditional Chinese Medicine	2	11	3	16
National Healthcare Security Admin[3]	0	8	7	15
State Drug Administration	1	7	4	12
National Development and Reform Commission	2	7	3	12
MO Industry and Information Technology	1	5	1	7
MO Science and Technology	0	0	3	3
Other departments combined[4]	0	8	1	9
**Singular/Joint releases**				
One department	17	48	20	85
Two departments	2	11	2	15
Three departments	0	3	0	3
Four or more combined	2	12	4	18

Supplementary Figure 1: Eligible policy documents by three time periods.[1]

Note:

1: MO: Ministry Of; admin: Administration.

2: The Chinese National Health Commission changed its title from "Ministry of Health" to "National Commission on Health and Family Planning" (17th March, 2013) and then to the current "National Health Commission" (13th March, 2018). Policies issued by these three different names were counted as one.

3: The national healthcare security admin was established in 2018, therefore its number was 0 in the 12th five-year.

4: Other ministries (n = 11): Ministry of Ecology and Environment, Ministry of Civil Affairs, Ministry of Housing and Urban-Rural Development, Ministry of Education, Ministry of Agriculture and Rural Affairs, General Administration of Sport, National Government Offices Administration, Development Research Centre of the State Council, Chinese Academy of Science, Chinese Academy of Social Science, and National Bureau of Statistics

WHO's six building blocks of health system	12th Five-Year Plan (2014-2015)	13th Five-Year Plan (2016-2020)	14th Five-Year Plan (2021-2023)	All
**Leadership and governance**	17	47	20	84
**Service delivery**	16	54	23	93
**Health workforce**	6	13	6	25
**Health information**	12	33	12	57
**Health financing**	17	62	22	101
**Medicines and technologies**	13	48	16	77

Supplementary Figure 2: Distribution of policies containing the WHO's six building blocks of health system over three time periods
